# The effects of ARID1A mutations on colorectal cancer and associations with PD‐L1 expression by stromal cells

**DOI:** 10.1002/cnr2.1420

**Published:** 2021-05-27

**Authors:** Tomohiro Kamori, Eiji Oki, Yoshifumi Shimada, Qingjiang Hu, Yuichi Hisamatsu, Koji Ando, Mototsugu Shimokawa, Toshifumi Wakai, Yoshinao Oda, Masaki Mori

**Affiliations:** ^1^ Department of Surgery and Science, Graduate School of Medical Sciences Kyushu University Fukuoka Japan; ^2^ Division of Digestive and General Surgery Niigata University Graduate School of Medical and Dental Sciences Niigata Japan; ^3^ Department of Biostatistics, Yamaguchi University Graduate School of Medicine Yamaguchi Japan; ^4^ Department of Anatomic Pathology, Pathological Sciences, Graduate School of Medical Sciences Kyushu University Fukuoka Japan

**Keywords:** ARID1A, colorectal cancer, microsatellite instability, PD‐L1

## Abstract

**Background:**

ARID1A is a component of the SWI/SNF complex, which controls the accessibility of proteins to DNA. *ARID1A* mutations are frequently observed in colorectal cancers (CRCs) and have been reported to be associated with high mutational burden and tumor PD‐L1 expression in vitro.

**Aim:**

To clarify the role of ARID1A mutation in CRC.

**Method and results:**

We used next generation sequencing (NGS) and immunohistochemistry on clinically obtained samples. A total of 201 CRC tissues from Niigata University and Niigata Center Hospital were processed by NGS using the CANCERPLEX panel. Immunohistochemistry for ARID1A, PD‐L1, MLH1, and MSH2 was performed on 66 propensity‐matched (33 microsatellite instability‐high [MSI‐H] and 33 microsatellite‐stable [MSS]) cases among 499 cases from Kyushu University. TCGA data were downloaded from cBioPortal. NGS showed significantly more mutations in ARID1A mutated CRCs (*p* = 0.01), and the trend was stronger for right‐sided CRCs than left‐sided. TCGA data confirmed these findings (*p* < 0.01). *BRAF* V600E and *ATM* mutations were also found at higher frequencies. Immunohistochemistry showed that 30% of MSI‐H CRCs had *ARID1A* loss, while this was true in only 6% of MSS CRCs. In both MSI‐H and MSS, PD‐L1 expression by stromal cells was enhanced in the *ARID1A*‐mutant groups (90% vs 39% in MSI‐H, 100% vs 26% in MSS).

**Conclusion:**

We found a higher mutational burden in *ARID1A*‐mutant CRCs, and IHC study showed that *ARID1A* loss was correlated with high PD‐L1 expression in stromal cells regardless of MSI status. These data support the idea that mutant *ARID1A* is a potential biomarker for CRCs.

## 1 INTRODUCTION

Despite recent progress in anticancer therapies, colorectal cancer (CRC) remains one of the leading causes of cancer‐related death worldwide.^1^ Fluorouracil plus either oxaliplatin or irinotecan with biological agents is standard‐of‐care for patients with progressive CRC, although it has limited efficacy.^1^ Since the emergence of immune checkpoint inhibitors (ICIs) that target the programmed death‐1 (PD‐1)/programmed death‐ligand 1 (PD‐L1) axis, there has been growing evidence that patients with DNA mismatch repair (dMMR)/microsatellite instability‐high (MSI‐H) CRC and those with a high tumor mutational burden (TMB) can greatly benefit from ICI treatment.^2^, ^3^ However, the overall response rate to ICIs was reported to be only around 36% in recent clinical trials among MSI‐H CRC patients.^4^, ^5^ This finding highlights the need for new immunotherapy biomarkers beyond MSI status and TMB.

AT‐rich interaction domain 1A (ARID1A) is a component of the SWI/SNF chromatin remodeling complex, which controls the accessibility of proteins to DNA.^6^ The *ARID1A* gene is commonly mutated in many types of cancer and is classified as a tumor suppressor.^7^ *ARID1A* is mutated in approximately 10% of CRC patients and is associated with medullary histology, *BRAF* V600E mutation, and MSI‐H status.^8^, ^9^, ^10^ Another study found that *ARID1A* loss or impaired ARID1A binding to MSH2 (a member of the MMR proteins) reduced MMR activity and increased the mutational load and PD‐L1 expression of tumor cells.^11^ In mice with *ARID1A*‐deficient ovarian cancer, the therapeutic effect of PD‐L1 inhibitors was greatly enhanced, suggesting ICI treatment could be beneficial for ARID1A‐impaired patients.^11^ Additionally, retrospective studies have reported relationships between *ARID1A* mutations and mutational load and with the immune environment, implying a clinical benefit from ICI in cancers harboring *ARID1A* mutations.^12^, ^13^, ^14^ These findings suggested that *ARID1A* mutations may not only cooperate with ICI treatment but could also have predictive value for ICI therapy.

In this study, we investigated the mutational status of *ARID1A* in CRCs using next generation sequencing (NGS) to reveal other co‐occurring cancer‐related mutations, the number of other mutations, and evaluate the relationship between PD‐L1 and ARID1A expression by immunohistochemistry (IHC) to test whether *ARID1A*‐mutant CRCs are more likely to express PD‐L1.

## 2 METHODS

### 2.1 Case selection

We collected two patient cohorts, one for NGS and the other for IHC analysis (Supplementary Figure 1). A total of 201 patients diagnosed with CRC according to the AJCC seventh edition^15^ who underwent curative surgery between 2009 and 2015 at Niigata University Medical and Dental Hospital or Niigata Cancer Center Hospital were enrolled for NGS (NGS cohort). Clinicopathological data including sex, age at surgery, tumor laterality (cecum to transverse colon is classified as right‐sided, and descending colon to rectum as left‐sided), histological type, pathological depth of invasion, lymph node metastasis, lymphatic invasion, vascular invasion, and pathological stage were collected.

For IHC, we retrospectively assessed 499 patients who underwent surgical resection for primary CRC between 1994 and 2015 at the Department of Surgery and Sciences, Graduate School of Kyushu University Hospital. The histological diagnoses were based on the AJCC seventh edition.^15^ Clinicopathological data were collected including sex, age at surgery, tumor laterality, histological type, lymphatic invasion, vascular invasion, and pathological stage.

### 2.2 Next generation sequencing

NGS data collection was performed at Niigata University, and the detailed procedures are explained in a previous report.^16^ Briefly, gDNA (50‐150 ng) was extracted from formalin fixed paraffin embedded samples, converted into libraries, and then enriched for the 415 genes in the CANCERPLEX (KEW Inc.; Cambridge, Massachusetts). CANCERPLEX is a clinically validated 415‐gene panel that is enriched for coding regions of genes with known associations to cancer. Sequencing was performed on the Illumina MiSeq and NextSeq platforms with an average sequencing depth of 500×. Genomic data were then processed through a proprietary bioinformatics platform and knowledge base to identify multiple classes of genomic abnormalities including single nucleotide substitutions (SNPs), small insertions/deletions (indels), and copy number variations (CNVs). A threshold of 10% allelic fraction was used for SNPs and indels, while thresholds of >2.5‐fold (gains) and 0.5‐fold (loss) were used for CNVs. To assess the somatic status of mutations in a tumor‐only setting, variants were deprioritized if they were present in a combination of the dbSNP, 1000 Genomes, or ExAC databases (at AF > 1%). Next, allele frequencies for each mutation were used to fit a model to determine whether the variant was likely germline heterozygous or somatic. Finally, the results underwent a manual review by a molecular pathologist, who validated the status of the variants (somatic vs possible germline). Based on published data and experience, this approach allows for the correct discrimination between germline and somatic variants in >99% of cases. TMB was determined by non‐synonymous SNPs present in the tumor that had a population frequency of <1% in the dbSNP and 1000 Genomes databases. The presence of *ARID1A* mutations and the mutational burden of The Cancer Genome Atlas (TCGA) cohort were downloaded from cBioPortal (https://www.cbioportal.org/).^17^, ^18^, ^19^

### 2.3 MSI analysis

MSI status was assessed in 499 patients of the Kyushu University cohort using fluorescent‐labeled primers and an automated DNA sequencer as described previously.^20^, ^21^, ^22^ Briefly, we amplified the microsatellite domain from cancerous and normal tissues by PCR. The fluorescent‐labeled polymerase chain reaction product was loaded onto an ABI 310 sequencer (Applied Biosystems, Foster City, California), and the data were analyzed using GeneScan software (Thermo Fisher Scientific, Waltham, Masasachusetts). MSI was determined based on the analysis of five reference markers (D2S123, D5S107, D10S197, D11S904, and D13S175). MSI‐H was defined as a replication error in at least two markers, MSI‐low (MSI‐L) was defined as a replication error in a single marker, and MSS was defined as no replication errors among the reference markers. Patients with MSS or MSI‐L were combined into the MSS group. MSI status was determined for all patients; 48 were classified as MSI‐H and the remaining 451 as MSS. Significant factors (sex and pathological stage) were used for propensity score analysis; as a result, 46 patients from each of the MSI‐H and MSS groups were selected. Ultimately, 33 MSI‐H and 33 MSS patients were included in the final histological analysis (IHC cohort, Supplementary Figure 1b).

### 2.4 Immunohistochemistry

**FIGURE 1:**
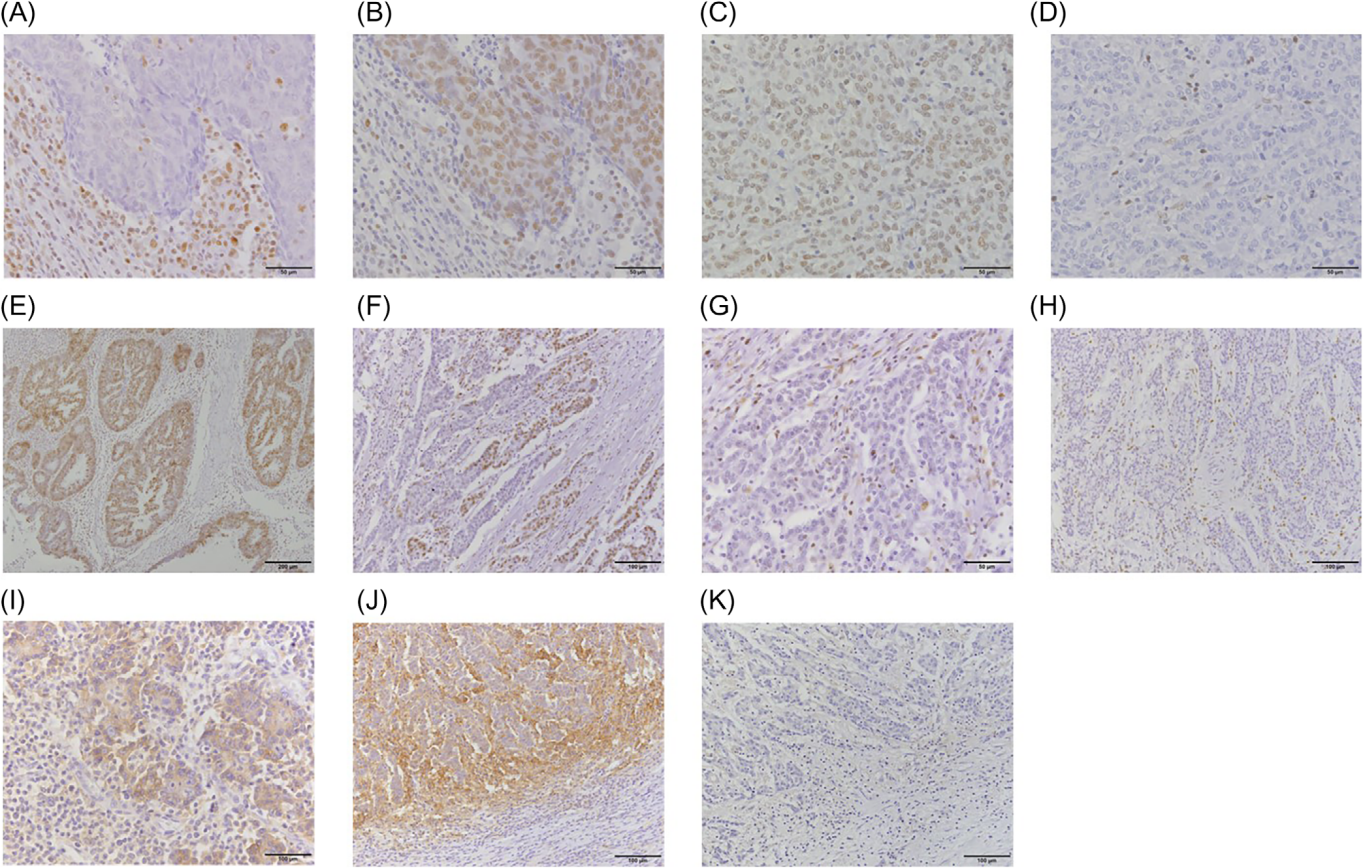
Immunohistochemical staining for MLH1, MSH2, AT‐rich interaction domain 1A(ARID1A), and programmed death‐ligand 1 (PD‐L1). (A) Negative MLH1 staining in the nuclei of tumor cells. (B) Positive MSH2 staining in the nuclei of both tumor and stromal cells. (C) Positive MLH1 staining in the nuclei of both cells. (D) Negative MSH2 staining in the nuclei of tumor cells. (E) Diffuse positive ARID1A staining in the nuclei of both cells. (F) Focal negative ARID1A staining in the nuclei of tumor cells. There are two separate regions, ARID1A‐negative on the right and ‐positive on the left. (G) High‐power magnification of (E), note that the nuclei of stromal cells in the ARID1A‐negative area are positive for ARID1A staining. (H) Diffuse negative ARID1A staining in the nuclei of tumor cells. (I) Positive membrane staining of PD‐L1 on the cytoplasm of tumor and stromal cells. (J) Positive membrane staining of PD‐L1 on the cytoplasm of stromal cells. (K) Negative PD‐L1 staining in both cells

We performed IHC using the universal immunoperoxidase polymer method (Envision‐kit; Dako‐Japan, Tokyo, Japan) for all available cases. Formalin‐fixed, paraffin‐embedded tissues were sectioned into 4‐μm slices, and then antigen retrieval was performed by boiling the slides in 10 mM sodium citrate (pH 6.0) or Target Retrieval Solution (Dako‐Japan). The primary antibodies and staining conditions are summarized in Supplementary Table 1. Anti‐mouse for MLH1 and MSH2 or anti‐rabbit for ARID1A and PD‐L1 IgG (DAKO‐Japan) were used as secondary antibodies. The stained slides were evaluated by Tomohiro Kamori (Figure 1).

The expression of MMR proteins (MLH1 and MSH2) was judged as “loss” when there was a complete absence of nuclear staining in neoplastic cells, while the surrounding non‐neoplastic cells consistently showed preserved nuclear staining (Figure 1A‐D).

**FIGURE 2:**
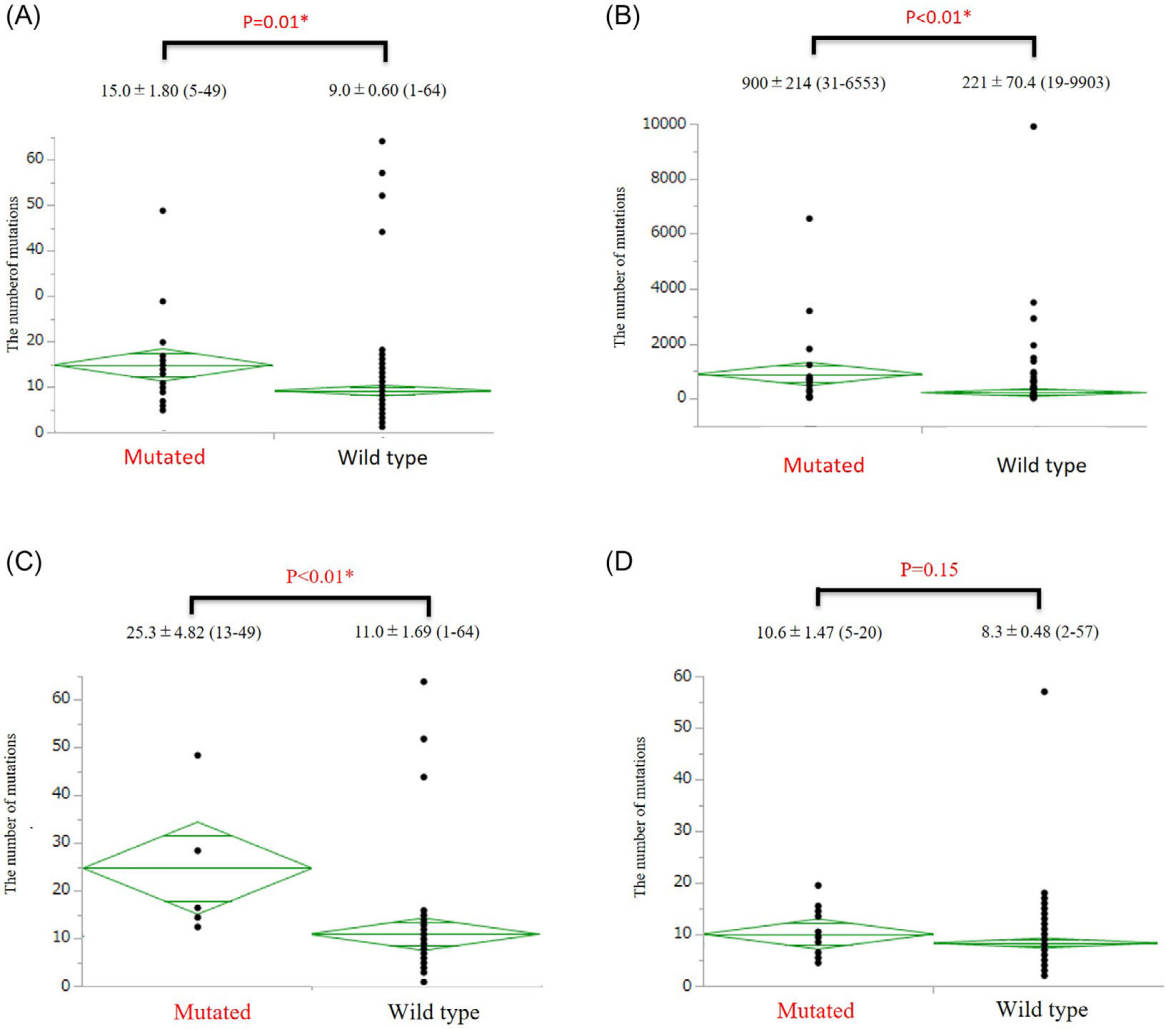
The number of mutations in the (A) next generation sequencing (NGS) and (B) The Cancer Genome Atlas (TCGA) cohort, as well as in the NGS cohort divided by (C) right‐ and (D) left‐sided disease. The graphs display the means and standard deviations (SD), minimum score and maximum score for each group. *Statistically significant difference between the *ARID1A*‐mutant and wild‐type groups (*p* < 0.05)

In our assessment of ARID1A, clear absence of staining in the nuclei of viable tumor tissue (away from necrotic areas) was considered “loss.” There were two types of ARID1A staining loss, total and focal loss. Both types were regarded as loss because at least part of the tumor was presumed to have *ARID1A* mutation (Figure 1E‐H). As a control, the presence of homogenous strong nuclear staining in background stromal fibroblasts, inflammatory cells, vascular endothelial cells, and normal epithelial cells was a prerequisite for assessing staining in the tumor.

PD‐1 was considered positive in tumor cells when membranous staining was evident in 1% or more of these cells; likewise, PD‐L1 was considered positive in stromal cells when membranous staining was observed in 1% or more of these cells (Figure 1I‐K). The 1% cutoff values are based on a previous study by Takada et al.^23^

### 2.5 Statistical analysis

We assessed statistical differences between groups using the Mann‐Whitney *U*‐test, the chi‐squared test, or Fisher's exact test. All calculations were performed using JMP software v13.0 (SAS Institute, Cary, North Carolina). *p*‐Values <0.05 were considered significant.

## 3 RESULTS

### 3.1 Clinicopathological characteristics and mutational status of the NGS group with ARID1A mutations

Clinicopathological features of the NGS group are summarized in Table 1. There were 22 patients with *ARID1A* mutations (10%) similar to previous reports.^9^, ^19^ We did not find any meaningful differences in age, sex, sidedness, tumor differentiation, lymphatic or venous invasion status, or pathological stage between the *ARID1A*‐mutated and wild‐type groups. Recently, the laterality of CRCs has been reported to determine their mutational features^24^; therefore, we divided the patients according to sidedness (Supplementary Tables 2 and 3). This revealed that tumor histological grade was significantly correlated with *ARID1A* mutation status in patients with right‐sided CRC, while this difference was not observed in left‐sided CRC patients. The mutational status of *ARID1A* and mutations that were observed more frequently in *ARID1A*‐mutant cases are listed in Supplementary Tables 4 and 5. *ARID1A* mutations were scattered throughout the *ARID1A* locus and there was no hotspot. Patients with *ARID1A* mutations were likely to have *ATM* (25%) or *BRAF* V600E (24%) mutations, which was similar to a previous report.^25^ All cases with both *BRAF* V600E and *ARID1A* mutations were right‐sided CRCs. The rare mutations observed in *ARID1A*‐mutant samples were thought to be due to the high mutation frequency in these patients. The most noteworthy findings from the sequencings results were the differences in TMB, which are depicted in Figure 2A. Patients with *ARID1A* mutations had a significantly greater number of mutations in the cancer‐associated sequenced by CANCERPLEX. This trend was also confirmed with TCGA database, which included 276 cases of whole exome sequencing (Figure 2B). In TCGA cohort, the mean number of mutations in *ARID1A*‐mutant CRCs was over 4‐fold greater than the number in *ARID1A* wild‐type CRCs. This feature was also affected by sidedness, as shown in Figure 2C,D. Only right‐sided CRCs with *ARID1A* mutations showed high mutational load compared with wild‐type CRCs. Left‐sided CRCs with *ARID1A* mutations also showed a slight tendency to have more mutations than wild‐type CRCs, although the difference was not significant. In summary, NGS analysis showed that right‐sided CRCs with *ARID1A* mutations were likely to have a distinct mutational signature (*BRAF* V600E and *ATM* mutations) and a higher TMB, which was compatible with previous results from in vitro studies.

**TABLE 1:** Clinicopathological features of *ARID1A*‐mutant and wild‐type patients in the NGS cohort

Clinicopathological features		ARID1A mutation				*p*‐Value
		Mutated		Wild type		
		*n* = 20	(10%)	*n* = 181	(90%)	
Age	<70	8	(40)	118	(65)	0.64
	70≦	12	(60)	63	(35)	
Sex	Male	12	(60)	105	(58)	0.75
	Female	8	(40)	76	(42)	
Sidedness	Right	6	(30)	49	(89)	0.78
	Left	14	(70)	132	(90)	
Histological grade	G1,G2	12	(60)	135	(75)	0.74
	G3	8	(40)	46	(25)	
ly	+	12	(60)	110	(61)	0.37
	−	8	(40)	71	(39)	
v	+	16	(80)	137	(90)	0.5
	−	4	(20)	44	(24)	
Stage	I‐IIIC	10	(50)	79	(44)	0.59
	IVA,IVB	10	(50)	103	(56)	

### 3.2 Clinicopathological characteristics and PD‐L1 expression in the IHC group

Clinicopathological features and IHC results of the IHC group are summarized in Table 2. We originally matched patients in the MSI‐H and MSS groups by sex and pathological stage; however, several patients could not be included in this study due to lack of specimens. Increased proportions of right‐sidedness and higher histological grade were observed in the MSI‐H group compared with the MSS group; these are previously known features of MSI‐H CRCs. There were also increased proportions of female patients and older patients (which are other features of MSI‐H CRCs), but these were not significant.

**TABLE 2:** Clinicopathological features and immunohistochemistry (IHC) results of microsatellite instability‐high (MSI‐H) and microsatellite stable (MSS) cases in the IHC cohort

Clinicopathological features and IHC	Status	MSI status				*p‐*Value
		High		Low/stable		
		*n* = 33	(%)	*n* = 33	(%)	
Age	<70	14	(42)	20	(61)	0.14
	70≦	19	(58)	13	(39)	
Sex	Male	11	(33)	16	(48)	0.21
	Female	22	(67)	17	(52)	
Sidedness	Right	25	(76)	5	(16)	<0.001*
	Left	8	(24)	27	(84)	
Histological grade	G1,G2	24	(73)	32	(97)	0.006*
	G3	9	(27)	1	(3)	
ly	+	12	(36)	10	(30)	0.37
	−	21	(64)	23	(70)	
v	+	6	(18)	13	(39)	0.06
	−	27	(82)	20	(61)	
Pathological stage	I‐IIC	26	(79)	23	(70)	0.40
	IIIA‐IVB	7	(21)	10	(30)	
ARID1A IHC	+	23	(70)	31	(94)	0.008*
	−	10	(30)	2	(6)	
PD‐L1 in tumor	+	8	(24)	0	(0)	0.002*
	−	25	(76)	33	(100)	
PD‐L1 in stroma	+	18	(55)	10	(30)	0.05*
	−	15	(45)	23	(70)	
MLH1 IHC	+	6	(18)	33	(100)	<0.001*
	−	27	(82)	0	(0)	
MSH2 IHC	+	30	(91)	33	(100)	0.04*
	−	3	(9)	0	(0)	

* Statistically significant difference (*p* < 0.05).

IHC results showed that ARID1A loss was much more prevalent among the MSI‐H group (30%) than the MSS group (6%). Most of the MSI‐H cases were due to impaired MLH1 (82%) and fewer showed MSH2 loss (9%) according to MMR protein IHC. The MSS group showed no irregular staining of either MMR proteins. PD‐L1 expression in both tumor (24%) and stromal (55%) cells was significantly enhanced in the MSI‐H group compared with the MSS group (0% and 30%, respectively). Detailed clinicopathological and IHC data of each group are shown in Tables 3 and 4. Interestingly, ARID1A loss by IHC was correlated with enhanced PD‐L1 expression by stromal cells in both the MSI‐H (90% in ARID1A‐deficient‐ and 10% in ARID1A‐present‐patients) and MSS (100% and 26%, respectively) groups. No other clinicopathological feature or IHC result shows an association with ARID1A status. In conclusion, impaired ARID1A staining was primarily observed in the MSI‐H group, but increased PD‐L1 expression by stromal cells was detected in ARID1A‐deficient patients from both the MSI‐H and MSS groups.

**TABLE 3:** Clinicopathological features and IHC results of the MSI‐H group of the IHC cohort

Clinicopathologic features and IHC	Status	ARID1A IHC				*p*‐Value
		Loss		Present		
		*n* = 10	(30%)	*n* = 23	(70%)	
Age	<70	3	(30)	11	(48)	0.34
	70≦	7	(70)	12	(52)	
Sex	Male	4	(40)	7	(30)	0.59
	Female	6	(60)	16	(70)	
Sidedness	Right	9	(90)	16	(70)	0.78
	Left	1	(10)	7	(30)	
Histological grade	G1,G2	8	(80)	16	(70)	0.74
	G3	2	(20)	7	(30)	
ly	+	4	(40)	8	(35)	0.78
	−	6	(60)	15	(65)	
v	+	2	(20)	4	(17)	0.86
	−	8	(80)	19	(83)	
Stage	I‐IIC	8	(80)	18	(78)	0.91
	IIIA‐IVB	2	(20)	5	(22)	
PD‐L1 in tumor	+	3	(30)	5	(22)	0.61
	−	7	(70)	18	(78)	
PD‐L1 in stroma	+	9	(90)	9	(39)	0.007*
	−	1	(10)	14	(61)	
MLH1 IHC	+	2	(20)	5	(22)	0.91
	−	8	(80)	18	(78)	
MSH2 IHC	+	9	(90)	21	(91)	0.90
	−	1	(10)	2	(9)	

* Statistically significant difference (*p* < 0.05).

**TABLE 4:** Clinicopathological features and IHC results of the MSS group of the IHC cohort

Clinicopathologic features and IHC	Status	ARID1A IHC				*p‐*Value
		Loss		Present		
		*n* = 2	(6%)	*n* = 31	(94%)	
Age	<70	1	(50)	19	(61)	0.75
	70≦	1	(50)	12	(39)	
Sex	Male	1	(50)	15	(48)	0.96
	Female	1	(50)	16	(52)	
Sidedness	Right	0	(0)	5	(17)	0.53
	Left	2	(100)	25	(83)	
Histological grade	G1,G2	2	(100)	30	(97)	0.79
	G3	0	(0)	1	(3)	
ly	+	0	(0)	10	(32)	0.33
	−	2	(100)	21	(68)	
v	+	1	(50)	12	(39)	0.75
	−	1	(50)	19	(61)	
Stage	I‐IIC	2	(100)	21	(68)	0.33
	IIIA‐IVB	0	(0)	10	(32)	
PD‐L1 in tumor	+	0	(0)	0	(0)	N/A
	−	2	(100)	31	(100)	
PD‐L1 in stroma	+	2	(100)	8	(26)	0.03*
	−	0	(0)	23	(74)	
MLH1 IHC	+	2	(100)	31	(100)	N/A
	−	0	(0)	0	(0)	
MSH2 IHC	+	2	(100)	31	(100)	N/A
	−	0	(0)	0	(0)	

* Statistically significant difference (*p* < 0.05).

## 4 DISCUSSION

We used two different cohorts to explore the impacts of *ARID1A* mutations on CRC and found that *ARID1A*‐mutated CRCs tended to have higher mutational loads and that ARID1A‐deficient CRCs were more likely to be accompanied by enhanced PD‐L1 expression by stromal cells. We used the CANCERPLEX panel, which can detect as many as 415 cancer‐related genes, to analyze the NGS group. This revealed two primary conclusions. One was that CRCs with *ARID1A* mutations were likely to co‐occur with *BRAF* V600E and *ATM* mutations, which are both clinically targetable with molecular therapies.^25^ The association between *ARID1A* mutations and the *BRAF* V600E mutation has already been reported,^8^ but co‐occurring *ATM* mutations are a new feature of *ARID1A*‐mutant CRCs. The *BRAF* V600E mutation is well known for its role in melanoma, and BRAF inhibitors such as dabrafenib have already been shown to be fairly effective in advanced CRCs with the *BRAF* V600E mutation in clinical studies.^25^ *ATM* is also a well‐known oncogene that regulates cell proliferation and DNA double strand break repair.^26^

The second conclusion from this analysis was that *ARID1A*‐mutant CRCs had a greater number of mutations compared with *ARID1A*‐wild type CRCs, and this relationship was stronger in right‐sided CRCs. We examined the number of co‐occurring mutations rather than mutational burden due to the lack of mutational burden data in our cohort. This is not a standard evaluation, and is one of the limitations of our study. However, we confirmed the same trend in TCGA cohort, and there are other reports in a large cohort study with over 40 000 cases that *ARID1A*‐mutated cancers including CRCs had higher mutational burdens than cancers without *ARID1A* mutations.^12^, ^14^ These data also reinforced the finding of higher mutation numbers in the *ARID1A*‐mutant CRCs in our cohort. High TMB is a good predictor for tumors that express high amounts of neoantigens, which recruit inflammatory cells including CD8‐positive T lymphocytes into the tumor microenvironment.^3^ These lymphocytes interact with the tumor cells via secreting IFNγ, and the tumor cells express PD‐L1 in turn to deactivate the lymphocytes and escape immune reactions.^27^ These findings were so critical to our study that we further determined an IHC evaluation of ARID1A and PD‐L1 expression to confirm the hypothesis that impaired ARID1A function enhances PD‐L1 expression by increasing the number of genetic mutations.

We evaluated 66 cases in the IHC group, of which 33 were MSI‐H and 33 were matched MSS cases. MSI‐H cases have been reported to have tendency toward being diagnosed in women and at earlier stages, so we matched these factors in the MSS group using propensity scoring. Increased proportions of older age, right‐sidedness, and high histological grade were seen, which have been repetitively reported for MSI‐H CRCs, ensuring the validity of our MSI‐H cohort. Loss of ARID1A staining was seen in 30% and 6% of MSI‐H and MSS cases, respectively. In both groups, loss of ARID1A staining was significantly correlated with elevated PD‐L1 expression in stromal cells. Several studies have investigated the consequences of PD‐L1 expression by stromal cells in CRC. Liu et al. reported that PD‐L1 expression was mainly observed in the stromal cells at the invasive front of MSI‐H CRCs.^28^ Korehisa et al. concluded that PD‐L1‐expressing stromal cells primarily consist of macrophages, especially tumor‐associated macrophages.^29^ According to those reports, the idea that immune cells, especially macrophages, at the invasive front of CRCs are the ones that express PD‐L1 and inactivate killer T‐cell function is feasible. In the CheckMate 142 clinical study, increased PD‐L1 expression by stromal cells was associated with a higher objective response rate; however, this finding was not significant.^5^ In our study, we found enhanced PD‐L1 expression only in stromal cells of CRCs with loss of ARID1A staining. Interestingly, PD‐L1 expression in stromal cells was also enhanced in the ARID1A‐impaired CRCs even with MSS status. Together, these findings suggest a model wherein CRCs with *ARID1A* mutations have a higher TMB, which leads to the recruitment of PD‐L1‐expressing immune cells (especially macrophages) to the invasive front, enabling the cancer cells to escape immune response. If PD‐1 or PD‐L1 blockade can break this signaling, ICIs should show efficacy in *ARID1A*‐mutant CRCs.

This study had several limitations. First, we applied different methods to two totally different cohorts. However, the association between *ARID1A* mutation and loss of its IHC staining was discussed in depth in a previous report,^30^ so there is evidence in the literature that CRCs with loss of ARID1A staining generally have *ARID1A* mutations, which supports the value of the IHC analysis in this study. The second is selection bias of the IHC group. We applied the propensity matching method to eliminate confounding factors (sex and pathological stage). CRCs with *ARID1A* mutations tended to have a higher histological grade and older age, which may interfere with the IHC results. The small sample size of our study is another limitation. A verification study using a validation cohort with a larger sample size will be necessary. A lack of standardized methods for PD‐L1 staining in CRCs is another limitation. We used the PD‐L1 clone 28‐8, which is a universally accepted antibody.^31^ PD‐L1 evaluation has been performed previously in the previous literature, but the 1% cut‐off value might be controversial. Finally, there was no case in our cohorts who used ICIs, as the ICIs were approved in 2019 for MSI‐H colorectal cancer in Japan. We investigated the outcome difference between ARID1A mutated and wild‐type patients in TCGA, NGS, or IHC groups. However, we did not find any difference in prognosis between ARID1A mutated and wild‐type patients.

In conclusion, this is the first report to reveal the close relationship between ARID1A and PD‐L1 expression by IHC in CRCs. We also confirmed co‐occurring *BRAF* V600E and *ATM* mutations in *ARID1A*‐mutant CRCs. Furthermore, ARID1A mutations were correlated with a higher mutation number, which may partly account for the enhanced PD‐L1 expression by stromal cells. Although this is a preliminary study, we have provided good evidence to indicate that *ARID1A* mutations in CRCs could be used to indicate ICI treatment. Recently, an association between *ARID1A* mutations and increased immune activity in gastrointestinal cancer was reported,^13^, ^14^ reinforcing our findings. Our finding that MSS CRCs with ARID1A loss also exhibited higher PD‐L1 expression by stromal cells may provide a new treatment option for MSS CRC patients. Along with the previous in vivo study that revealed the role of ARID1A mutations in the increased mutational load and antitumor effects of ICIs,^11^ using clinically obtained specimens, this study reinforced the idea that *ARID1A* mutations can be biomarkers for using ICIs in CRC patients.

## CONFLICT OF INTEREST

Eiji Oki reports receiving honoraria for lecturing from Chugai Pharmaceutical Co., Ltd. Taiho Pharmaceutical Co., Ltd., Bayer Yakuhin Japan, Eli Lilly, Yakult Honsha Co., Ltd., Takeda Pharmaceutical Co., Ltd., ONO Pharmaceutical Co., Ltd. and Merck Biopharma Co., Ltd., outside the submitted work.

## AUTHOR CONTRIBUTIONS

*Conceptualization*, T.K., E.O., Y.S., Q.H., Y.H., K.A., T.W., Y.O. and M.M.; *Methodology*, T.K., E.O., Y.S., T.W. and Y.O.; *Investigation*, T.K., E.O., and S.Y.; *Formal Analysis*, T.K., E.O., Q.H, Y.H., K.A. and M.S.; *Resources*, T.K., E.O., Y.S., T.W., Y.O. and M.M.; *Writing—Original Draft*, T.K. and E.O.; *Writing—Review & Editing*, T.K., E.O., T.W., Y.O. and M.M.; *Supervision*, T.W, Y.O and M.M.; *Project administration*, M.M.

## ETHICS STATEMENT

This study was approved by the institutional review board of Kyushu University Fukuoka and Niigata University Niigata, Japan (permission number: 30‐367) and conformed to the tenets of the Declaration of Helsinki. Informed consent was waived by the institutional review board owing to the retrospective nature of the study.

## Supporting information


**Supplementary Figure 1** Case selection charts for the (a) NGS and (b) IHC cohorts

**Supplementary Table 1** Clones, companies, and dilution conditions for the primary antibodies used in this study
**Supplementary Table 2**. Clinicopathological features of right‐sided CRCs with or without *ARID1A* mutations in the NGS cohort. *Statistically significant difference (*p* < 0.05)
**Supplementary Table 3**. Clinicopathological features of left‐sided CRCs with or without *ARID1A* mutations in the NGS cohort
**Supplementary Table 4**. Detailed mutational status of *ARID1A* in the NGS cohort
**Supplementary Table 5**. Co‐occurring mutations that were seen at significantly higher frequencies in the *ARID1A*‐mutant NGS cohort. *Statistically significant difference (*p* < 0.05)

## Data Availability

The result of Figure 2B is in whole based upon data generated by the TCGA Research Network: https://www.cancer.gov/tcga. The other data that support the findings of this study are available on request from the corresponding author. The data are not publicly available due to privacy or ethical restrictions.

## References

[1] Saltz LB . Value in colorectal cancer treatment: where it is lacking, and why. Cancer J. 2016;22(3):232‐235. 27341604 10.1097/PPO.0000000000000194 PMC5543411

[2] Le DT , Uram JN , Wang H , et al. PD‐1 blockade in tumors with mismatch‐repair deficiency. N Engl J Med. 2015;372(26):2509‐2520. 26028255 10.1056/NEJMoa1500596 PMC4481136

[3] Cristescu R , Mogg R , Ayers M , et al. Pan‐tumor genomic biomarkers for PD‐1 checkpoint blockade– based immunotherapy. Science. 2018;362(6411):eaar3593. 30309915 10.1126/science.aar3593 PMC6718162

[4] Le DT , Kim TW , Van Cutsem E , et al. Phase II open‐label study of Pembrolizumab in treatment‐refractory, microsatellite instability‐high/mismatch repair‐deficient metastatic colorectal cancer: KEYNOTE‐164. J Clin Oncol. 2020;38(1):11‐19. 31725351 10.1200/JCO.19.02107 PMC7031958

[5] Overman MJ , McDermott R , Leach JL , et al. Nivolumab in patients with metastatic DNA mismatch repair‐deficient or microsatellite instability‐high colorectal cancer (CheckMate 142): an open‐label, multicentre, phase 2 study. Lancet Oncol. 2017;18:1182‐1191. 28734759 10.1016/S1470-2045(17)30422-9 PMC6207072

[6] Clapier CR , Iwasa J , Cairns BR , Peterson CL . Mechanisms of action and regulation of ATP‐dependent chromatin‐remodelling complexes. Nat Rev Mol Cell Biol. 2017;18:407‐422. 28512350 10.1038/nrm.2017.26 PMC8127953

[7] Nagl NG Jr , Wang X , Patsialou A , Van Scoy M , Moran E . Distinct mammalian SWI/SNF chromatin remodeling complexes with opposing roles in cell‐cycle control. EMBO J. 2007;26(3):752‐763. 17255939 10.1038/sj.emboj.7601541 PMC1794396

[8] Chou A , Toon CW , Clarkson A , et al. Loss of ARID1A expression in colorectal carcinoma is strongly associated with mismatch repair deficiency. Hum Pathol. 2014;45(8):1697‐1703. 24925223 10.1016/j.humpath.2014.04.009

[9] Ye J , Zhou Y , Weiser MR , et al. Immunohistochemical detection of ARID1A in colorectalcarcinoma: loss of staining is associated with sporadic microsatellite unstable tumors with medullary histology and high TNM stage. Hum Pathol. 2014;45:2430‐2436. 25311944 10.1016/j.humpath.2014.08.007

[10] Kim Y‐S , Jeong H , Choi J‐W , Hwa Eun O , Lee J‐H . Unique characteristics of ARID1A mutation and protein level in gastric and colorectal cancer: a meta‐analysis. Saudi J Gastroenterol. 2017;23(5):268‐274. 28937020 10.4103/sjg.SJG_184_17 PMC5625362

[11] Shen J , Zhenlin J , Zhao W , et al. ARID1A deficiency promotes mutability and potentiates therapeutic antitumor immunity unleashed by immune checkpoint blockade. Nat Med. 2018;24(5):556‐562. 29736026 10.1038/s41591-018-0012-z PMC6076433

[12] Jiang T , Chen X , Chunxia S , Ren S , Zhou C . Pan‐cancer analysis of ARID1A alterations as biomarkers for immunotherapy outcomes. J Cancer. 2020;11(4):776‐780. 31949479 10.7150/jca.41296 PMC6959029

[13] Lin L , Li M , Jiang Z , Wang X . ARID1A mutations are associated with increased immune activity in gastrointestinal cancer. Cell. 2019;8(7):678. 10.3390/cells8070678 PMC6678467 31277418

[14] Tokunaga R , Xiu J , Goldberg RM , et al. The impact of ARID1A mutation on molecular characteristics in colorectal cancer. Eur J Cancer. 2020;140:119‐129. 33080474 10.1016/j.ejca.2020.09.006 PMC8009046

[15] Edge SB , Byrd DR , Compton CC , Fritz AG , Greene FL , Andy Trotti III . AJCC Cancer Staging Manual. 7th ed. New York: Springer; 2010:143‐159.

[16] Nagahashi M , Wakai T , Shimada Y , et al. Genomic landscape of colorectal cancer in Japan: clinical implications of comprehensive genomic sequencing for precision medicine. Genome Med. 2016;8:136. 28007036 10.1186/s13073-016-0387-8 PMC5180401

[17] Ethan Cerami , Jianjiong Gao , Ugur Dogrusoz , et al. The cBio cancer genomics portal: an open platform for exploring multidimensional cancer genomics data. Cancer Discov. 2012;2(5):401–4 22588877 10.1158/2159-8290.CD-12-0095 PMC3956037

[18] Gao J , Aksoy BA , Dogrusoz U , et al. Integrative analysis of complex cancer genomics and clinical profiles using the cBioPortal. Sci Signal. 2013;6(269):l1. 10.1126/scisignal.2004088 PMC4160307 23550210

[19] Cancer Genome Atlas Network . Comprehensive molecular characterization of human colon and Rectal cancer. Nature. 2012;487(7407):330‐337. 22810696 10.1038/nature11252 PMC3401966

[20] Toh Y , Oki E , Oda S , et al. An integrated microsatellite length analysis using an automated fluorescent DNA sequencer. Cancer Res. 1996;56:2688‐2691. 8665494

[21] Oda S , Oki E , Maehara Y , Sugimachi K . Precise assessment of microsatellite instability using high resolution fluorescent microsatellite analysis. Nucleic Acids Res. 1997;25:3415‐3420. 9254697 10.1093/nar/25.17.3415 PMC146902

[22] Oki E , Oda S , Maehara Y , Sugimachi K . Mutated genespecific phenotypes of dinucleotide repeat instability in human colorectal carcinoma cell lines deficient in DNA mismatch repair. Oncogene. 1999;18:2143‐2147. 10321739 10.1038/sj.onc.1202583

[23] Takada K , Okamoto T , Shoji F , et al. Clinical significance of PD‐L1 protein expression in surgically resected primary lung adenocarcinoma. J Thorac Oncol. 2016;11:1879‐1890. 27346415 10.1016/j.jtho.2016.06.006

[24] Kamran SC , Clark JW , Zheng H , et al. Primary tumor sidedness is an independent prognostic marker for survival in metastatic colorectal cancer: results from a large retrospective cohort with mutational analysis. Cancer Med. 2018;7(7):2934‐2942. 10.1002/cam4.1558 PMC6051212 29771009

[25] Corcoran RB , André T , Atreya CE , et al. Combined BRAF, EGFR, and MEK inhibition in patients with BRAFV600E‐mutant colorectal cancer. Cancer Discov. 2018;8(4):428‐443. 29431699 10.1158/2159-8290.CD-17-1226 PMC5882509

[26] Sriramulu S , Ramachandran M , Subramanian S , et al. A review on role of ATM gene in hereditary transfer of colorectal cancer. Acta Biomed. 2018;89(4):463‐469. 10.23750/abm.v89i4.6095 PMC6502098 30657113

[27] Garcia‐Diaz A , Shin DS , Moreno BH , et al. Interferon receptor signaling pathways regulating PD‐L1 and PD‐L2 expression. Cell Rep. 2017;19(6):1189‐1201. 28494868 10.1016/j.celrep.2017.04.031 PMC6420824

[28] Liu S , Gӧnen M , Stadler ZK , Weiser MR , et al. Cellular localization of PD‐L1 expression in mismatch‐repairdeficient and proficient colorectal carcinomas. Modern Pathol. 2019;32:110‐121. 10.1038/s41379-018-0114-7 PMC6309293 30166615

[29] Korehisa S , Oki E , Iimori M , et al. Clinical significance of programmed cell death‐ligand 1 expression and the immune microenvironment at the invasive front of colorectal cancers with high microsatellite instability. Int J Cancer. 2018;142(4):822‐832. 29044503 10.1002/ijc.31107

[30] Khalique S , Naidoo K , Attygalle AD , et al. Optimised ARID1A immunohistochemistry is an accurate predictor of ARID1A mutational status in gynaecological cancers. J Pathol Clin Res. 2018;4(3):154‐166. 29659191 10.1002/cjp2.103 PMC6065117

[31] Büttner R , Gosney JR , Skov BG , et al. Programmed death‐ligand 1 immunohistochemistry testing: a review of analytical assays and clinical implementation in non‐small‐cell lung cancer. J Clin Oncol. 2017;35(34):3867‐3876. 29053400 10.1200/JCO.2017.74.7642

