# Supplementary figures and images for: The effects of ARID1A mutations on colorectal cancer and associations with PD‐L1 expression by stromal cells
Source: Cancer Rep (Hoboken). 2021 May 27;5(1):e1420. doi: 10.1002/cnr2.1420 (PMC8789618; doi:10.1002/cnr2.1420)

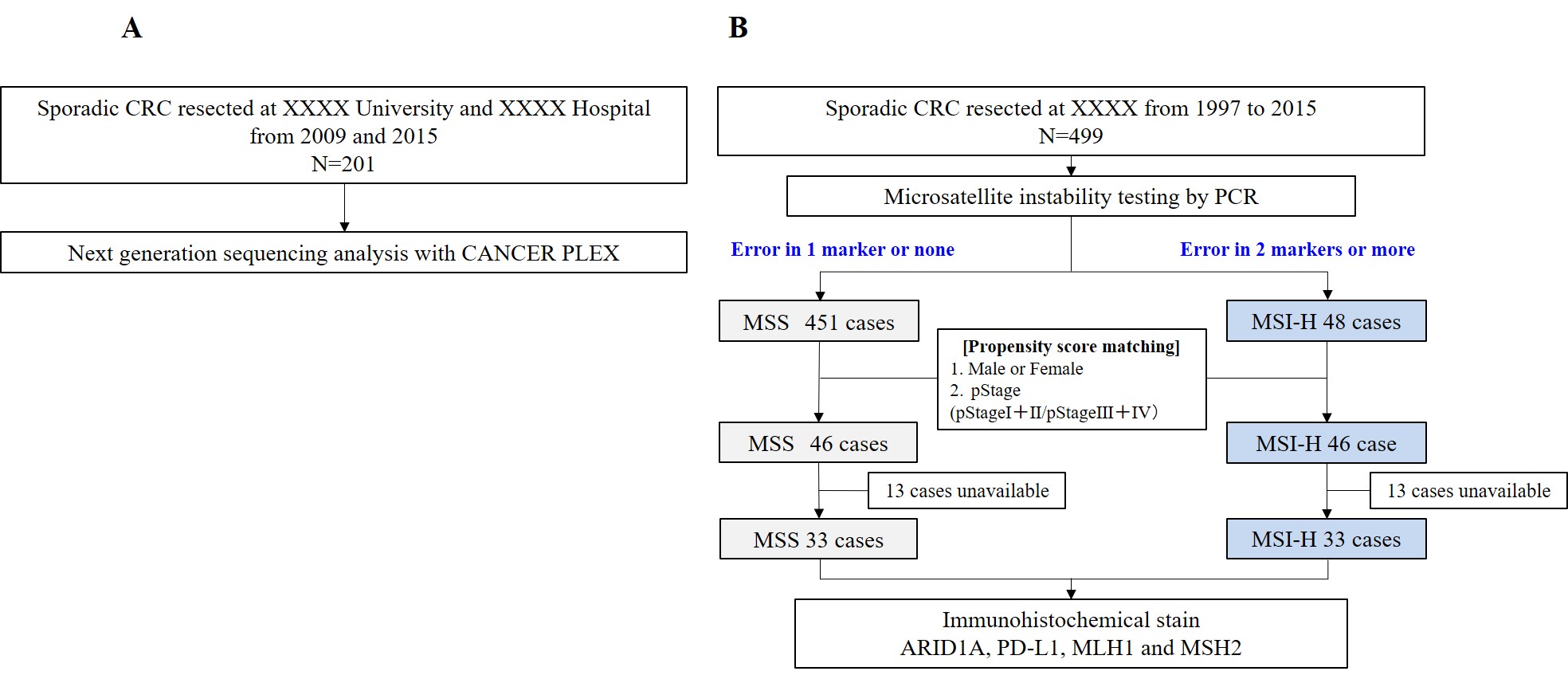

Supplement: Supplementary file 1 — Supplementary Figure 1 Case selection charts for the (a) NGS and (b) IHC cohorts [file CNR2-5-e1420-s002.jpg]
