# Supplementary material for: The effects of ARID1A mutations on colorectal cancer and associations with PD‐L1 expression by stromal cells
Source: Cancer Rep (Hoboken). 2021 May 27;5(1):e1420. doi: 10.1002/cnr2.1420 (PMC8789618; doi:10.1002/cnr2.1420)
Supplement: Supplementary file 2 — Supplementary Table 1 Clones, companies, and dilution conditions for the primary antibodies used in this study Supplementary Table 2. Clinicopathological features of right‐sided CRCs with or without ARID1A mutations in the NGS cohort. *Statistically significant difference (p < 0.05) Supplementary Table 3. Clinicopathological features of left‐sided CRCs with or without ARID1A mutations in the NGS cohort Supplementary Table 4. Detailed mutational status of ARID1A in the NGS cohort Supplementary Table 5. Co‐occurring mutations that were seen at significantly higher frequencies in the ARID1A‐mutant NGS cohort. *Statistically significant difference (p < 0.05) [file CNR2-5-e1420-s001.docx]

Supplementary Table 1. Clones, companies, and dilution conditions for the primary antibodies used in this study.

| Antibody | Clone | Company | Dilution | Secondary antibody |
| --- | --- | --- | --- | --- |
| MLH1 | G168-15 | BD Pharmingen | 1:50 | Mouse |
| MSH2 | 79H11 | Leica | 1:120 | Mouse |
| ARID1A | Polyclonal | SIGMA | 1:500 | Rabbit |
| PD-L1 | 8-28 | Abcam | 1:480 | Rabbit |

Supplementary Table 2. Clinicopathological features of right-sided CRCs with or without *ARID1A* mutations in the NGS cohort. *Statistically significant difference (p<0.05).

| Clinicopathological features | | ARID1A mutation | | | | P value |
| --- | --- | --- | --- | --- | --- | --- |
|  |  | Mutated | | Wild type | |  |
|  |  | n=6 | (11%) | n=49 | (89%) |  |
| age | <70 | 4 | (67) | 20 | (41) | 0.23 |
|  | 70≦ | 2 | (6) | 29 | (59) |  |
| sex | male | 3 | (50) | 23 | (47) | 0.89 |
|  | female | 3 | (50) | 26 | (53) |  |
| Histological grade | G1,G2 | 0 | (0) | 30 | (61) | <0.01* |
|  | G3 | 6 | (100) | 19 | (39) |  |
| ly | + | 6 | (100) | 31 | (63) | 0.07* |
|  | - | 0 | (0) | 18 | (37) |  |
| v | + | 5 | (83) | 40 | (82) | 0.92 |
|  | - | 1 | (17) | 9 | (18) |  |
| Stage | I-IIIC | 2 | (33) | 19 | (39) | 0.8 |
|  | IVA,IVB | 4 | (67) | 30 | (61) |  |

Supplementary Table 3. Clinicopathological features of left-sided CRCs with or without *ARID1A* mutations in the NGS cohort.

| Clinicopathologcail features | | ARID1A mutation | | | | P value |
| --- | --- | --- | --- | --- | --- | --- |
|  |  | Mutated | | Wild type | |  |
|  |  | n=14 | (10%) | n=132 | (90%) |  |
| age | <70 | 10 | (71) | 89 | (67) | 0.76 |
|  | 70≦ | 4 | (29) | 43 | (33) |  |
| sex | male | 9 | (64) | 82 | (62) | 0.87 |
|  | female | 5 | (36) | 50 | (38) |  |
| Histological grade | G1,G2 | 12 | (86) | 105 | (80) | 0.58 |
|  | G3 | 2 | (14) | 27 | (20) |  |
| ly | + | 6 | (43) | 79 | (60) | 0.22 |
|  | - | 8 | (57) | 53 | (40) |  |
| v | + | 11 | (79) | 97 | (73) | 0.68 |
|  | - | 3 | (21) | 35 | (27) |  |
| Stage | I-IIIC | 3 | (21) | 65 | (49) | 0.05 |
|  | IVA,IVB | 11 | (79) | 67 | (51) |  |

Supplementary Table 4. Detailed mutational status of *ARID1A* in the NGS cohort.

| Mutational status of ARID1A |
| --- |
| P7fs |
| S90G |
| V132fs, I1173fs |
| G328D |
| 339_343del |
| S711X, Q932X |
| H782L |
| Y823X |
| N1070fs, H1336R, Q1512X |
| R1335X |
| H1336R |
| Q1399P |
| R1446X |
| P1567fs |
| R1721X |
| G1847fs |
| L2008fs |
| E2078X |
| P2139fs |
| Deletion |

Supplementary Table 5. Co-occurring mutations that were seen at significantly higher frequencies in the *ARID1A*-mutant NGS cohort. *Statistically significant difference (p<0.05).

| Gene | Status | ARID1A status | | | | | P value |
| --- | --- | --- | --- | --- | --- | --- | --- |
|  |  | Mutated | |  | Wild type | |  |
|  |  | n=20 | (%) |  | n=181 | (%) |  |
| ATM | Mutated | 5 | (25) |  | 16 | (9) | 0.03* |
|  | Wild | 15 | (75) |  | 165 | (91) |  |
| BRAF | Mutated | 7 | (35) |  | 17 | (9) | <0.01* |
|  | Wild | 13 | (65) |  | 164 | (91) |  |
| BRAF V600E | Mutated | 4 | (24) |  | 9 | (5) | 0.04* |
|  | Wild | 13 | (76) |  | 164 | (95) |  |
| CUX1 | Mutated | 4 | (20) |  | 1 | (1) | <0.01* |
|  | Wild | 16 | (80) |  | 180 | (99) |  |
| EPHA5 | Mutated | 2 | (10) |  | 0 | (0) | <0.01* |
|  | Wild | 18 | (90) |  | 181 | (100) |  |
| FANCL | Mutated | 1 | (5) |  | 0 | (0) | 0.03* |
|  | Wild | 19 | (95) |  | 181 | (100) |  |
| GNA11 | Mutated | 2 | (10) |  | 2 | (1) | 0.04* |
|  | Wild | 18 | (90) |  | 179 | (99) |  |
| HNF1A | Mutated | 1 | (5) |  | 0 | (0) | 0.03* |
|  | Wild | 19 | (95) |  | 181 | (100) |  |
| JAK1 | Mutated | 2 | (10) |  | 2 | (1) | 0.04* |
|  | Wild | 18 | (90) |  | 179 | (99) |  |
| KAT6A | Mutated | 1 | (5) |  | 0 | (0) | 0.03* |
|  | Wild | 19 | (95) |  | 181 | (100) |  |
| MED12 | Mutated | 2 | (10) |  | 0 | (0) | <0.01* |
|  | Wild | 18 | (90) |  | 181 | (100) |  |
| MITF | Mutated | 2 | (10) |  | 0 | (0) | <0.01* |
|  | Wild | 18 | (90) |  | 181 | (100) |  |
| PPARG | Mutated | 1 | (5) |  | 0 | (0) | 0.03* |
|  | Wild | 19 | (95) |  | 181 | (100) |  |
| RUNX1 | Mutated | 2 | (10) |  | 1 | (1) | 0.01* |
|  | Wild | 18 | (90) |  | 180 | (99) |  |
| USP9X | Mutated | 2 | (10) |  | 0 | (0) | <0.01* |
|  | Wild | 18 | (90) |  | 181 | (100) |  |
